# In Search of Factors Negatively Affecting Vaccine Immunity to Pertussis in Preschool Children before the Administration of the First Booster

**DOI:** 10.3390/ijerph15071432

**Published:** 2018-07-06

**Authors:** Anna Bednarek, Anna Bodajko-Grochowska, Barbara Hasiec, Robert Klepacz, Katarzyna Szczekala, Danuta Zarzycka, Andrzej Emeryk

**Affiliations:** 1 Department of Pediatric Nursing, Medical University of Lublin, Lublin 20-059, Poland; zarzycka.danuta@wp.pl; 2 Department of Pulmonary Diseases and Children Rheumatology, Medical University of Lublin, Lublin 20-059, Poland; andziii@poczta.onet.pl (A.B.-G.); emerykandrzej@gmail.com (A.E.); 3 Department of Infectious Diseases of Children, Independent Public Provincial Hospital of Jan of God, Lublin 20-400, Poland; bhasiec@wp.pl; 4 Department of Clinical Pathomorphology, Medical University of Lublin, Lublin 20-059, Poland; rklepacz@wp.pl; 5 Department of Foreign Languages, I Faculty of Medicine with Dentistry Division, Medical University of Lublin, Lublin 20-059, Poland; kasiasz12@wp.pl

**Keywords:** negatively affecting factors, pertussis, preschool children, vaccine immunity

## Abstract

*Introduction*: The top priority for active immunoprophylaxis of pertussis is the immunisation of infants as they can sometimes develop severe multiple-organ complications. *Objectives*: The aim of the work is the identification of factors negatively affecting vaccine immunity to pertussis in preschool children prior to the administration of the first booster. *Patients and Methods*: The research was conducted on 352 children from 4.5 to 5.9 years of age who were hospitalised in the University Children’s Hospital in Lublin (Poland) from 1 January 2012 to 31 December 2015. The children taking part in the study had been administered all the mandatory vaccines from their birth to the age of 2 or 2.5 years old according to the Polish Immunisation Program 2008–2009. The immunoenzymatic method ELISA (enzyme-linked immunosorbent assay) was applied to assess vaccine immunity to tetanus, diphtheria, pertussis, *Haemophilus influenzae type b* (Hib), *poliomyelitis* (IPV), mumps, rubella and measles. The level of vaccine antibodies to hepatitis type B was determined chemilumiscently. *Results*: The protective antibody titre was not found in 41 (11.65%) children before the administration of the booster. To verify the collective impact of parameters analysed on antibody titre to pertussis, the Generalized Linear Model (GLZ) was used. Gender, type of vaccine, asthma, Hib and mumps antibody titres have been shown to be predictors of vaccine immunity to pertussis. *Conclusions*: Immunomodulation considered on the example of titre of IgG antibody to pertussis can serve as a useful model of the assessment of development of acquired immunity after mandatory vaccinations.

## 1. Introduction

Paediatric immunisation is an essential element of specific primary prevention and maturing of mechanisms of humoural immunity. The development of immunological competence commences in intrauterine life. From birth on, the system matures due mainly to antigen stimulation. A significant increase in immunoglobulin concentration (IgG) occurs consistently from the second half year of an infant to the age of 15 only then it reaches values similar to adults’ ones [1,2].

Protective vaccination plays a major role in the development of specific active humoural immunity. On the other hand, both maternal malnutrition and infantile low birth weight can have a negative influence on the immune system performance in later life. Hygienic lifestyle, reasonable diet and the frequency and course of infections also play a vital role [3].

Pertussis is a highly contagious respiratory disease caused by Gram-negative bacterium *Bordetella pertussis*. Being ill with pertussis alone provides long-term immunity, though it does not exclude contracting the disease again [4]. The disease occurs in all age groups; however, its course is severest in the youngest infants because of their immature respiratory and immune systems as well as, due to their age, the lack of complete or even preliminary vaccination. Therefore, the top priority for pertussis active immunoprophylaxis is immunisation of infants who can develop severe complications such as pneumonia, apnoea, convulsions, encephalopathy or even death [5,6].

Due to common vaccination with whole-cell DTP (or DTwP) vaccines containing a suspension of *Bordetella pertussis*, the incidence of pertussis has diminished all over the world. However, the use of these preparations is associated with common local and systemic adverse reactions, which has led to the replacement of DTwP vaccines with acellular DTaP ones in developed countries [7,8,9].

Although a high percentage of the population has been vaccinated, pertussis is still an essential problem of public health and epidemic focuses are registered in European countries. The main reason for a recurrence of the disease is the rapid loss of immunity, namely 7–20 years after being ill with the disease and 5–10 years after vaccination with DTaP preparations. [10,11,12].

Comparing the incidence of pertussis in many countries is complicated because of differences of epidemiological supervision systems and diagnostics, the application of various vaccines as well as different vaccination schedules [13]. The incidence of pertussis is still growing in the developed countries, though immunoprophylaxis is commonly available.

In the European Union (EU), there is a multi-fold increase in the incidence of pertussis solely in adults and a relatively low death rate in infants younger than 1 year old. However, in the USA from 2004–2010, severe complications were noted including several dozen deaths from pertussis in infants ≤3 months old [14,15,16,17].

In Poland over the last years, the incidence of pertussis has also grown. The latest epidemiological data show that in 2012 a more than threefold increase in the disease was reported in comparison with the data in 2011. The highest incidence is found in teenagers and young adults who were administered the last dose of the vaccine in their childhood [18,19,20,21].

In response to a deteriorating worldwide epidemiological situation caused by the incidence of pertussis, the Global Pertussis Initiative (GPI) updated guidelines concerning pertussis vaccination in 2005. The need for the reinforcement of three strategies was noted, that is, vaccinating the mother, the new-born and the family members or people in the closest environment (the cocoon strategy). The GPI estimated that routine vaccination of teenagers along with cocooning can decrease the incidence of pertussis by 50% in infants. Since 2006 this strategy has been advised to be introduced in all countries that have appropriate resources. Simultaneously, the recommendations on DTaP vaccination issued by the Advisory Committee on Immunization Practices (ACIP) also included individuals ≥65 years of age, especially those who may have close contact with infants [19,22,23,24].

Since 2016 the Polish Immunisation Programme (PIP) has included four obligatory doses of primary pertussis vaccination, one booster and one additional booster at the age of 14 years old. For infants up to 16–18 months of age, it prescribes an entire cycle of primary vaccination with combined DTwP preparations (whole-cell vaccine) or DTaP (acellular vaccine), which is three doses and one booster. The PIP refunds acellular pertussis vaccines solely for infants and children of high clinical risk. DTaP booster vaccines are administered to 6-year-olds and dTap vaccines, which are preparations with a decreased amount of acellular pertussis component, are administered to 14-year-olds. In accordance with the PIP, vaccination against pertussis is given in a cycle of other obligatory primary vaccinations that most often include HBV, *poliomyelitis* (IPV) and *Haemophilus influenzae type b* (Hib) vaccinations as well as measles, mumps and rubella (MMR) vaccinations and additionally, since 2017, *Streptococcus pneumoniae* vaccination.

The aim of the study is to identify factors negatively affecting vaccine immunity to pertussis in preschool children prior to the administration of the first booster.

## 2. Materials and Methods

### 2.1. Study Group of Children

The study was conducted on 352 Caucasian children of 4.5–5.9 years of age who had been hospitalised in the University Children’s Hospital in Lublin (Poland) from 1 January 2012 to 31 December 2015. Around 40% of the children had been newly diagnosed with early childhood asthma and treated according to the 2012 criteria of GINA (Global Initiative for Asthma), that is low-dose inhaled corticosteroids (ICS) taken permanently and short-acting β2-mimetic (SABA) taken temporarily [25]. The mothers of the children researched had not been vaccinated against pertussis in pregnancy. The main inclusion criteria to be met were male and female genders, having had all the mandatory vaccinations from birth to 2–2.5 years of age according to the Polish Immunisation Programme of 2008–2009. Other criteria included at period of least 3 years following the last dose of vaccine against pertussis and lack of immunodeficiency in the children.

The exclusion criteria were as follows: hospital stay due to an infectious disease (including pertussis), the occurrence of an infection or immunosuppressive treatment within a two-month period preceding the investigation of antibodies in the serum.

Medical data regarding the children were collected from updated hospital documentation and immunisation cards and based on interviews with their parents.

Among 360 children meeting the eligibility criteria at the beginning of the study, 8 individuals were excluded, including 2 children because of the lack of appropriate medical immunisation records, also 2 children due to incomplete obligatory protective vaccinations and 4 whose parents declined consent to their child’s immunological blood test. Ultimately, the analyses were conducted on 352 children who participated until the end of the study.

The Bioethics Committee of the Medical University of Lublin (Poland) approved the research (no. KE-0254/176/2011). The study was performed within research project DS.514/2012-2015, financed by the Medical University of Lublin. The children who qualified for the study were pre-schoolers; thus, verbal consent from the children and written informed consent from both of their parents was gathered prior to the commencement of research procedures.

A total of 176 children were immunized with the following vaccines: 3 basic doses of monovalent hepatitis B vaccines (HBV) 24 h after birth then at the ages of 2 and 7 months; 2 basic doses and one booster of inactivated poliomyelitis vaccine (IPV) at the ages of 4, 6 and 16 months; 3 basic doses and one booster of Haemophilus influenzae type b (Hib) at the ages of 2, 4, 6 and 16 months; and three basic doses and one booster of three-ingredient whole-cell combined diphtheria tetanus and pertussis vaccine (DTwP) at the ages of 2, 4, 6 and 16 months.

The remaining 176 children were administered three doses and one booster of six-ingredient viral and bacterial HBV-DTaP-IPV-Hib (Infanrix hexa) vaccine with acellular pertussis component at the ages of 2, 4, 6 and 16 months.

**Figure 1:**
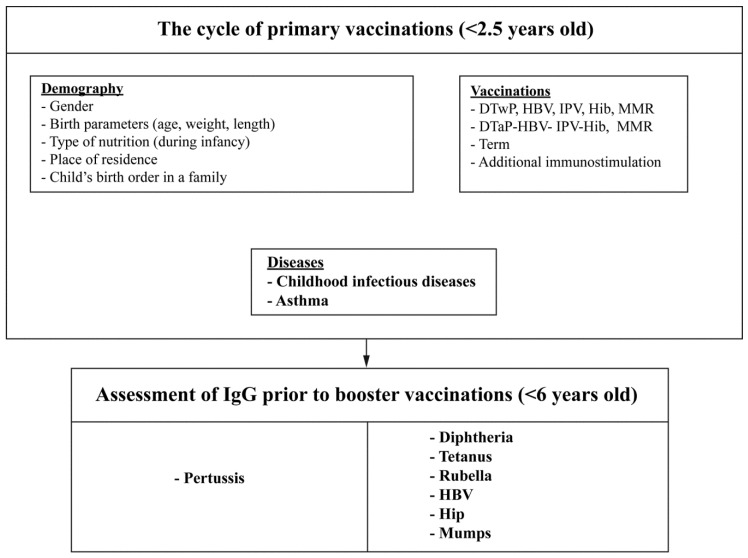
Research algorithm—parameters analysed included in the schedule of mandatory vaccinations.

All the children had been vaccinated with one dose of three-component combined MMR vaccine at the age of 13 months. The study was carried out based on the algorithm shown in Figure 1.

### 2.2. Serological Analysis

For serological analysis, blood specimens were collected from the fasting children on the morning of day 2 of their hospital stay. A total of 4.7 mL of venous blood was collected by means of the S-Monovette® 4.9 mL system with clotting activator (Sarstedt, Nümbrecht, Germany). The blood was then centrifuged at 300× *g* for 10 min at 4 °C. The sera achieved were collected into 1.5-mL polyethylene Eppendorf test tubes and stored frozen at −20 °C until the analysis. None of the samples showed a trace of haemolysis.

The immunoenzymatic method ELISA was applied to assess vaccine immunity to tetanus, diphtheria, pertussis, *Haemophilus influenzae type B* (Hib), poliomyelitis (IPV), mumps, rubella and measles. Certified diagnostic tests were used: the concentration of IgG antibodies was measured using a kit by IBL International GmbH (Hamburg, Germany) and the concentration of tetanus antibodies was checked with an ELISA Automation/Cortez Diagnostics, Calabasas, CA, USA). The investigation was performed with the *VICTOR*™ *X3 Multilabel* Plate Reader by Perkin Elmer, (Waltham, MA, USA). The data were obtained on the microplate reader using WorkOut 2.0 software. The level of vaccine antibodies to hepatitis type B was determined chemilumiscently by means of the ADVIA Centaur XP Immunoassay System from Siemens Healthineers (Erlangen, Germany).

The measurement of the level of IgG antibodies was carried out according to the manufacturers’ instructions. Protective values of IgG antibody titres for the vaccinations analysed were obtained consistently with the manufacturers’ procedures and taking into account the period from the last dose of rudimental vaccinations: 1.0 IU/mL for anti-diphtheria and anti-tetanus IgG; 10 IU/mL for anti-pertussis IgG against pertussis toxin (PT) and filamentous hemagglutinin (FHA) of *Bordetella pertussis*; 12.5 mIU/mL for anti-HBV IgG; 12 mIU/mL for anti-polio virus type 1, 2 and 3 IgG; 12 U/mL for anti-mumps IgG; 12 IU/mL for anti-rubella IgG; 1.0 µg/mL for anti-Hib IgG and 300 mIU/mL for anti-measles IgG.

### 2.3. Statistical Analysis

Statistical analysis of continuous variables was presented as arithmetic means and standard deviations (SDs). The distribution of the continuous variables was verified by means of the Shapiro-Wilk test. Depending on the type of distribution, statistical significance of the differences was verified using the Student *t*-test or the Mann-Whitney *U*-test. The distributions of discrete variables were presented as numbers and percentages and their intergroup comparisons were verified applying the chi-squared test and Fisher’s exact test. Furthermore, the analysis of logistic regression was conducted to determine the proportion of chances (ORs) to coexisting characteristic factors for a lower level of antibodies after pertussis vaccinations and their 95% confidence intervals (CIs). Additionally, the logistic regression analysis (the GLZ Multiple Regression, Probit Model, Best subsets) was applied to determine associations between the features analysed and the categorised titres of pertussis antibodies. The Wald test was used for the determination of the significance of explanatory variables (predictors) in the model assumed. All the calculations were performed using Statistica 12 (StaSoft, Tulsa, OK, USA). Significance threshold for all the tests was established as *p* < 0.05.

## 3. Results

No statistically significant difference was found in basic anthropometric measurements of the children researched, in regard to both birth and current measurements, depending on pertussis titre (Table 1).

It was statistically significant that a low pertussis antibody titre was found more frequently in the children with newly diagnosed asthma who were on immunostimulating drugs and had been administered whole-cell pertussis vaccine (DTwP). Other demographic and medical features analysed were not associated statistically significantly with the categorised pertussis antibody titre (Table 2).

Except for vaccination against Hib, where it was statistically significant that low antibody titre more frequently occurred in the children with low pertussis antibody titre, titres of antibodies to other vaccinations (HBV, mumps, rubella, diphtheria) were not correlated with the pertussis antibody titre. Protective antibody titres were found in 100% following vaccinations against poliomyelitis (≥12 U/mL) and measles (≥300 mlU/mL) (Table 3).

To verify collective impact of the parameters analysed on titres of antibody to pertussis, the Model GLZ Multiple Regression (Probit Model, Best subsets) was created. The model showed that gender, type of vaccine, asthma, titres of antibodies to Hib and mumps are predictors of titres of antibody to pertussis (Table 4).

**Figure 2:**
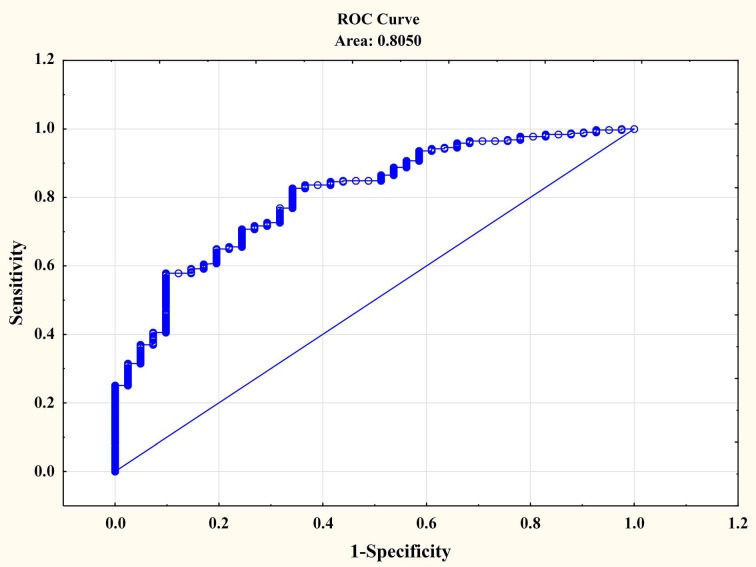
Goodness of Fit in the model applied based on the ROC curve.

The quality of classification for the model applied was evaluated based on a receiver operating characteristic curve (ROC). The value of the field under the curve (Area = 0.80) indicated appropriate model classification (Figure 2).

In the model of the analysis applied, the Wald test enabled the determination of pertussis vaccine immunity predictors for the population studied. What is crucial, particularly from the clinical point of view, is the fact that among these predictors a type of vaccine and the “group” were found.

## 4. Discussion

Conditions of appropriate immune response include genetic features of the individual being vaccinated, their age, gender, race, presence of maternal antibodies, overall clinical condition as well as exposure to differentiated environmental modifiers. With age, some disturbances of the immune system performance occur and its functions decline, which is of essential significance to the health and life of adults and ageing individuals. The consequence of the genetic remodelling is higher susceptibility of those individuals to infectious diseases and a diminished effectiveness of vaccinations [26,27].

Protection against infectious diseases in infants is of great significance and it is achieved due to maternal IgG antibodies which permeate the barrier of the placenta and later IgA antibodies in breast milk produced by the mother in response to an infection. Maternal breast milk is rich in substances that protect an infant when a bacterial infection occurs and support the child’s developing immune system. Nevertheless, the substances are not proved to show sufficient protective functions in the case of some infectious diseases, including pertussis. The aforementioned results of the research also suggest that the type of nutrition of infants does not affect titres of vaccine-induced antibodies [1,2,3].

Response to vaccination also depends on gender. Females, of different age groups, produce more specific antibodies and some adverse reactions are noted more frequently after the administration of vaccines. Individual variables in response to vaccination also result from polymorphism of single nucleotides involved in specific and non-specific response to bacterial vaccines, which plays an important role in the intensification of cellular response to the acellular vaccine against pertussis [28,29]. Gender did not determine vaccine-induced response against pertussis in this research.

The immune system of an infant or a young child can recognize and react to millions of antigens at the same time. The number of antigens administered in vaccines constitute an insignificant percentage of the value. Numerous studies have shown that immune response is the same to multi-component and uncombined vaccines. Therefore, the simultaneous administration of several vaccines does not adversely affect immune response. Moreover, the production of specific antibodies and creation of memory immune cells is similar to the separate administration of vaccines. Immune response to vaccination also depends on the clinical condition of the vaccinated individual and concomitant diseases. Those factors can be a reason for lack of response to vaccination and increased catabolism of antibodies as well as their loss via the digestive system and kidneys [30,31,32].

According to the WHO, coverage of pertussis vaccination in the world is around 86%. The widespread application of pertussis vaccinations, both acellular (aP) and whole-cell (wP), has turned out to be highly favourable for the decline in the frequency of the disease worldwide, although despite the implementation of protective vaccinations, the disease is still not fully controlled [19]. In our research, 88% of the infants (*n* = 311) reached antibody titre indicating immunity after the completion of the three-year-period of rudimental pertussis vaccination. Immunisation of pregnant women against pertussis is an effective strategy for preventing the disease in the youngest infants (<3 months old). Currently, immunisation of pregnant women with the acellular vaccine against pertussis is required between the 28th and 38th weeks of pregnancy, in the hope that the mothers will transmit vaccine-induced antibodies via the placenta to their children and in this way the children will be effectively protected against the disease during early childhood. However, other research reports also suggest that the presence of maternal antibodies may weaken children’s immune response to vaccinations given to infants after birth [33,34,35,36]. None of the mothers participating in our research were vaccinated against *Bordetella pertussis* during pregnancy.

According to the WHO, coverage of pertussis vaccination in the world is around 86%. The widespread application of pertussis vaccinations, both acellular (aP) and whole-cell (wP), has turned out to be highly favourable for the decline in the frequency of the disease worldwide, although despite the implementation of protective vaccinations, the disease is still not fully controlled [19]. In our research, 88% of the infants (*n* = 311) reached antibody titre indicating immunity after the completion of the three-year-period of rudimental pertussis vaccination. Immunisation of pregnant women against pertussis is an effective strategy for preventing the disease in the youngest infants (<3 months old). Currently, immunisation of pregnant women with the acellular vaccine against pertussis is required between the 28th and 38th weeks of pregnancy, in the hope that the mothers will transmit vaccine-induced antibodies via the placenta to their children and in this way the children will be effectively protected against the disease during early childhood. However, other research reports also suggest that the presence of maternal antibodies may weaken children’s immune response to vaccinations given to infants after birth [33,34,35,36]. None of the mothers participating in our research were vaccinated against *Bordetella pertussis* during pregnancy.

Research has indicated that pertussis vaccines, both whole-cell and acellular ones, do not provide long-term protection that atrophies, though boosters are given. The Italian prospective randomised study by Salmaso et al. showed that protection lasted 6 years after basic vaccination had been covered with two different acellular vaccines containing 3 antigens of *Bordetella pertussis*. They ascertained that vaccine is 76–86% effective during the first 6 years of life and reaches the range of 76–86% [28]. However, maintaining protection 5 years following vaccination was described by Gustaffson et al., who evaluated the long-term effectiveness of the DTaP vaccine administered to Swedish children at 3, 5 and 12 months of age. Increased prevalence of pertussis was observed in children aged 6–8 years [12].

In turn, Witt et al. evaluated the period of protection after DTaP vaccination in children during a pertussis epidemic in 2010 in California, USA. A total of 171 individuals with laboratory-confirmed pertussis were identified. Decreased actual effectiveness of the vaccine (24%) was noted in the group of 8–12-year-olds, whereas in 2–7-year-olds and 13–18-year-olds it was 41% and 79% respectively. A reason for the greater incidence of pertussis in 8–12-year-old children was acknowledged to be atrophy of vaccine-induced immunity following the administration of DTaP booster to pre-schoolers [34].

The effectiveness of anti-pertussis preparations should be considered from the perspective of individual protection against becoming ill with the disease, the period of protection and the possibility of surviving transmission of the pathogen, so as to produce collective vaccine-induced immunity. The protective titre of antibodies lasts a relatively short time in comparison with expectations, as well as other vaccinations [35].

In an American clinical controlled study encompassing the period from January 2006 to June 2011, post-vaccine protection was evaluated in school children vaccinated with DTaP at the age of 47–84 months old. The authors compared a group of 277 children who had pertussis confirmed by the Polymerase Chain Reaction (PCR) method with two control groups: 3318 children with a negative score of the PCR method for checking the presence of *Bordetella pertussis* and 6086 children selected according to some particular features of the population. It was said that with every year after giving the fifth dose of DTaP the risk of contracting pertussis increased on average 42% (OR: 1.42; 95% Cl: 1.21–1.66). The highest percentage of positive scores with the PCR method was obtained by children aged 8–11 years old who had been given five doses of DTaP vaccine when they were 4–6 years old [36,37,38,39].

The possibility of atrophy of DTaP vaccine-induced immunity can be explained by the considerable percentage of the youth and adults suffering from pertussis observed over the last decades. Other research also suggests that the risk of pertussis is increased in teenagers whose vaccination schedule in infancy included solely acellular vaccines against pertussis in comparison with adolescents who had been given ≥1 dose of whole-cell vaccine against pertussis [40].

Protection against pertussis in children up to 5 years old of age who were given complete rudimental vaccinations is well documented in literature. However, Fisher et al. conducted research in Canada and showed that a change of whole-cell vaccine into DTaP vaccine was connected with increased incidence of pertussis in children immunised only with DTaP.

The effectiveness of acellular vaccines can be lower that of some whole-cell vaccines, which was pointed out in an Australian study. Sheridan et al. compared the incidence of pertussis in children immunised with three whole-cell vaccines against diphtheria, tetanus and pertussis (DTwP), with the incidence in children who were given three doses of DTaP vaccines within primary immunisation. During the 10 years following primary immunisation, both during epidemics and years without epidemics, the risk of getting pertussis was lower in children vaccinated with DTwP than in those vaccinated with DTaP. Similar results were achieved in an American study encompassing children born at the time when whole-cell vaccines were replaced with acellular ones [14,30,40,41,42]. In this study, Liko et al. demonstrated that children immunised with DTaP contracted pertussis more frequently than children getting whole-cell vaccines within primary immunisation. However, clinical trials also showed that whole-cell vaccines against pertussis (DTwP) can significantly differ in terms of effectiveness and the immunogenicity of some of them ranged from 30–50% [9,43]. In our research, a high post-vaccine titre was significantly more frequently observed in the group of children vaccinated with combined DTaP preparations.

The main source of infection of pertussis for infants and young children is adults. Infants ill with pertussis confirmed by laboratory tests were investigated for 20 months in 4 countries. In 70% of the cases a contact with an infected adult was confirmed and in 50% of the cases an active infection of pertussis in one of the parents was noted. The aforementioned results show how important the administration of boosters (dTpa) to adults (pregnant mothers, fathers, relatives aged ≥65) is if they spend much time with their children, particularly those who have incomplete immunisation against pertussis [40,44,45].

In Poland, the implementation of immunisation of children against pertussis has been high (94–96%), though Poland is one of not many countries still using whole-cell (DTP) preparations within the PIP while acellular (DTaP) vaccines are available. Most developed countries resigned from whole-cell vaccines in favour of safety of children [4,36,46].

In our research, some of the children did not have protective titres of antibodies against pertussis about 3 years after the complete cycle of rudimental immunisation (4 doses; *n* = 41). However, it is worth mentioning that all the parents of the infants studied along with other adult individuals from their closest environment had not been additionally vaccinated against pertussis. In Poland, supplementary pertussis immunisation of adult members of family is not commonly used. Lack of a protective titre of antibodies also concerned other rudimental vaccinations such as diphtheria (*n* = 28), tetanus (*n* = 18), HBV (*n* = 11), mumps (*n* = 10) and rubella (*n* = 6). Only a low titre of antibodies against Hib was significantly associated with a low level of antibodies against pertussis.

Inability of a vaccinated individual to produce and/or maintain a satisfactory level of antibodies following the administration of rudimental vaccinations or boosters affects about 2–10% of the healthy who have been vaccinated and depends on both vaccine-related factors and on individual features of the organism of a vaccinated person. In most cases, the thorough determination of immunological causes, clinical consequences as well as the issue of lack of response is antigen- specific or determined by other factors frequently unfeasible to determine [8,15].

Before puberty asthma is more common in boys than in girls. Many aspects of differences in gender in the development and progress of asthma require further research. Both hormonal and genetic factors have an impact on that. Some polymorphisms are known to be particularly associated with asthma in females. Moreover, the influence of hormones on asthma and atopy needs researching with reference to innate and adaptive immunity in both sexes [47].

On the basis of research, specific IgG4 induced by allergen specific immunotherapy (SIT) has been found to be an immunological marker associated with the occurrence of clinical tolerance. Levels of IgG4 were analysed sevenfold (at the following weeks: 0, 5, 10, 25, 52, 104 and 156). After this 156-week cycle of the subcutaneous administration of SIT *Dermatophagoides pteronyssinus* (Der p) in 226 children and 109 adults with allergic rhinitis and/or asthma, there were no differences found between females and males at any time point. Therefore, the authors concluded that children are more sensitive to SIT showing clinical improvement and IgG4 higher levels production during shorter periods of SIT in comparison to adults. The increase of specific IgG4 for Der P reflects children’s specific immune response to SIT vaccine [48].

In many developed countries, a change of lifestyle and nutrition constitutes a great health problem that can exert a considerable influence on the immune system and post-immunisation antibody titre [41,42,43]. It is difficult to enumerate all possible causes of the lack of protective titre of antibodies against pertussis and other diseases covered by vaccinations in early childhood. Nonetheless, the model we have adopted involved the determination of parameters that negatively affect IgG value and so shape vaccine immunity to pertussis.

## 5. Conclusions

Immunomodulation considered in the example of titre of IgG antibodies to pertussis can constitute a valuable model for the assessment of adaptive immunity development after the completion of rudimental vaccinations. The features established such as a type of vaccine, the immune system stimulation via immunostimulating drugs and immune diseases (asthma) determine the maintenance of appropriate titre of antibodies prior to the administration of the first booster.

## Figures and Tables

**Table 1:** Pertussis antibody titre and anthropometric measurements.

Feature	Pertussis ≥10 U/mL (+) *			Pertussis <10 U/mL (−)			*p*
	*n*	Mean	SD	*n*	Mean	SD	
Birth weight (g)	311	3416.05	462.72	41	3432.75	465.81	0.7699
Current weight (kg)	311	20.73	3.55	41	20.61	4.35	0.8034
Birth body length (cm)	311	55.13	3.75	41	54.55	4.38	0.3288
Current height (cm)	311	117.86	4.54	41	118.46	5.29	0.5468

* The value of antibody titre that indicates vaccine immunity to pertussis was shown in units of measurement consistent with the manufacturers’ guidelines. The lowest level protecting against contracting the disease in pre-schoolers was depicted as “+” according to the manufacturers’ instructions. SD: standard deviation.

**Table 2:** Pertussis antibody titre and selected demographic, medical and vaccinological parameters.

Feature		Pertussis ≥10 U/mL (+) *n* = 311		Pertussis <10 U/mL (−) *n* = 41		*p*	OR	95% CI	
		*n*	%	*n*	%				
Gender	Male	172	55.31	27	65.85	0.2003	1.56	0.79	3.09
	Female	139	44.69	14	34.15				
Birth age (week)	<37 (32–36)	41	13.18	5	12.20	0.5466	0.91	0.34	2.47
	≥37	270	86.82	36	87.80				
First child in family	Yes	86	27.65	9	21.95	0.2823	1.36	0.62	2.97
	No	225	72.35	32	78.05				
Age *	<Me	133	42.77	17	41.46	0.8741	0.95	0.49	1.84
	>Me	178	57.23	24	58.54				
Place of residence	Town	221	71.06	27	65.85	0.4921	0.79	0.39	1.57
	Countryside	90	28.94	14	34.15				
Suspected asthma	Yes	142	45.66	34	82.93	<0.001	5.78	2.49	1.44
	No	169	54.34	7	17.07				
Suffered infectious diseases **	Yes	51	16.40	6	14.63	0.4909	1.14	0.46	2.86
	No	260	83.60	35	85.37				
Taking immunostimulating drugs ***	Yes	150	48.23	29	70.73	<0.01	0.39	0.19	0.78
	No	161	51.77	12	29.27				
Type of nutrition at the age of 0–6 months old	Breast milk + evaporated milk formula	33	10.61	5	12.20	0.7195			
	Breast milk	200	64.31	28	68.29				
	evaporated milk formula	78	25.08	8	19.51				
Type of immunisation (vaccine) from birth to 2–2.5 years old	Uncombined (DTwP, HBV, IPV, Hib, MMR)	147	47.27	29	70.73	< 0.01	2.70	1.33	5.48
	Combined (DTaP-HBV-IPV-Hib, MMR)	164	52.73	12	29.27				
Promptness of vaccinations	Yes	230	73.95	29	70.73	0.6599	1.17	0.57	2.41
	No	81	26.05	12	29.27				

* Median; Me = 5.2 yr. ** Chickenpox (*n* = 29), rotavirus infection (*n* = 16). *** Ribomunyl (*n* = 15), Ismigen (*n* = 12), Broncho-Vaxom (*n* = 32) given one course of each vaccine. DTwP: whole-cell combined diphtheria, tetanus and pertussis vaccine; HBV: hepatitis B vaccine; IPV: inactivated poliomyelitis vaccine; Hib: Haemophilus influenzae type b vaccine; MMR: measles, mumps and rubella vaccine.

**Table 3:** Concentration of pertussis antibody and other antibody titres.

Type of Vaccination and Protective Antibody Titre		Pertussis ≥10 U/mL (+) *n* = 311		Pertussis <10 U/mL (−) *n* = 41		*p*	OR	95% CI	
		*n*	%	*n*	%				
**Diphtheria**	≥1 IU/mL (+)	140	45.02	13	31.71	0.1061	0.57	0.28	1.14
	<1 IU/mL	171	54.98	28	68.29				
**Tetanus**	≥1 IU/mL (+)	221	71.06	23	56.10	0.0508	0.52	0.27	1.01
	<1 IU/mL	90	28.94	18	43.90				
**HBV**	>12.5 mlU/mL (+)	249	80.06	30	73.17	0.3061	0.68	0.32	1.43
	<12.5 mlU/mL	62	19.94	11	26.83				
**Hib**	≥1 µg/mL (+)	284	91.32	31	75.61	<0.01	0.29	0.13	0.67
	<1 µg/mL	27	8.68	10	24.39				
**Mumps**	≥12 U/mL (+)	253	81.35	31	75.61	0.381	0.71	0.33	1.53
	<12 U/mL	58	18.65	10	24.39				
**Rubella**	≥12 lU/mL (+)	277	89.07	35	85.37	0.3151	0.72	0.28	1.83
	<12 lU/mL	34	10.93	6	14.63				

**Table 4:** Generalised Linear Model (GLZ) Multiple Regression (Probit Model, Best subsets).

Category of Analysis	Wald	*p*
Age A	0.64	0.4253
Gender	3.99	0.0459
Place of residence	1.86	0.1729
Childhood infectious diseases undergone	0.08	0.7716
Taking immunestimulating preparations	0.00	0.9643
Birth age	0.02	0.8840
Type of nutrition at the age of 0-6 months old	0.49	0.4826
Type of vaccine	8.22	0.0041
Promptness of vaccinations	0.30	0.5853
Group (children tentatively diagnosed with asthma)	12.93	0.0003
Hepatitis B	1.56	0.2113
Diphtheria	1.70	0.1924
Tetanus	0.08	0.7817
Hib	6.17	0.0130
Mumps	4.78	0.0288
